# With Impressions Chosen from Another Time: Core Technologies and Debitage Production at the Lower Palaeolithic Site of Notarchirico (670–695 ka; layers F to I2)

**DOI:** 10.1007/s41982-023-00154-y

**Published:** 2023-09-05

**Authors:** Marco Carpentieri, Marie-Hélène Moncel, Giacomo Eramo, Marta Arzarello

**Affiliations:** 1 Dipartimento Studi Umanistici, Università Degli Studi Di Ferrara, C.So Ercole I d’Este 32, 44121 Ferrara, Italy; 2 UMR 7194 HNHP (MNHN-CNRS-UPVD), Département Homme Et Environnement, Muséum National d’Histoire Naturelle, 1 Rue René Panhard, 75013 Paris, France; 3 Dipartimento Di Scienze Della Terra E Geoambientali, Università Degli Studi Di Bari Aldo Moro, 70125 Bari, Italy

**Keywords:** Lower Palaeolithic, Core technologies, Acheulean

## Abstract

The earliest evidence of bifaces in western Europe is dated to the initial phase of the Middle Pleistocene (la Noira, Notarchirico, Moulin Quignon, 700–670 ka), with the findings of Barranc de la Boella (1.0–0.9 Ma) considered to be an earlier local evolution. No transition assemblages are recorded during this time frame, and the “abrupt” appearance of bifaces during this time frame is associated with significant cognitive shifts in human technological behaviours (Acheulean techno-complex). The new investigations conducted at the site of Notarchirico unearthed 30 ka of repeated human occupation (695–670 ka, layers F-I2) during MIS 17, with evidence of bifacial tools in layer G (680 ka) and F along with other heavy-duty implements (LCTs, pebble tools, etc.). Massive production of *debitage* products realised on local raw materials collected in situ through simple and efficient core technologies characterises a large part of the lithic assemblage with a high ratio of diversified light-duty tools, including modified chert nodules. Despite core and flake assemblages being a recurrent trait of Lower Pleistocene contexts, the increase in retouched implements recorded at the onset of the Middle Pleistocene has been considered a significant technological shift. The technological analysis of the *debitage* products presented in this work highlights recurrent and systematic technological behaviours of the hominins of Notarchirico—who proved to efficiently overcome the raw materials dimensional constraints—even in the layers without bifaces. This may shed light on the meaning of cultural and behavioural innovation that the Acheulean techno-complex is thought to bring over Europe. It is plausible that given the substantial homogeneity of the lithic strategies within the sequence of Notarchirico, which only the “introduction” of the bifaces in the upper layers seems to interrupt, a supposed behavioural or cultural change in the site might have already occurred in the lowermost portion of the sequence. In this work, we evaluate the degree of change—if any—from a technological perspective by analysing the *debitage* reduction sequences.

## Introduction

**Fig. 1:**
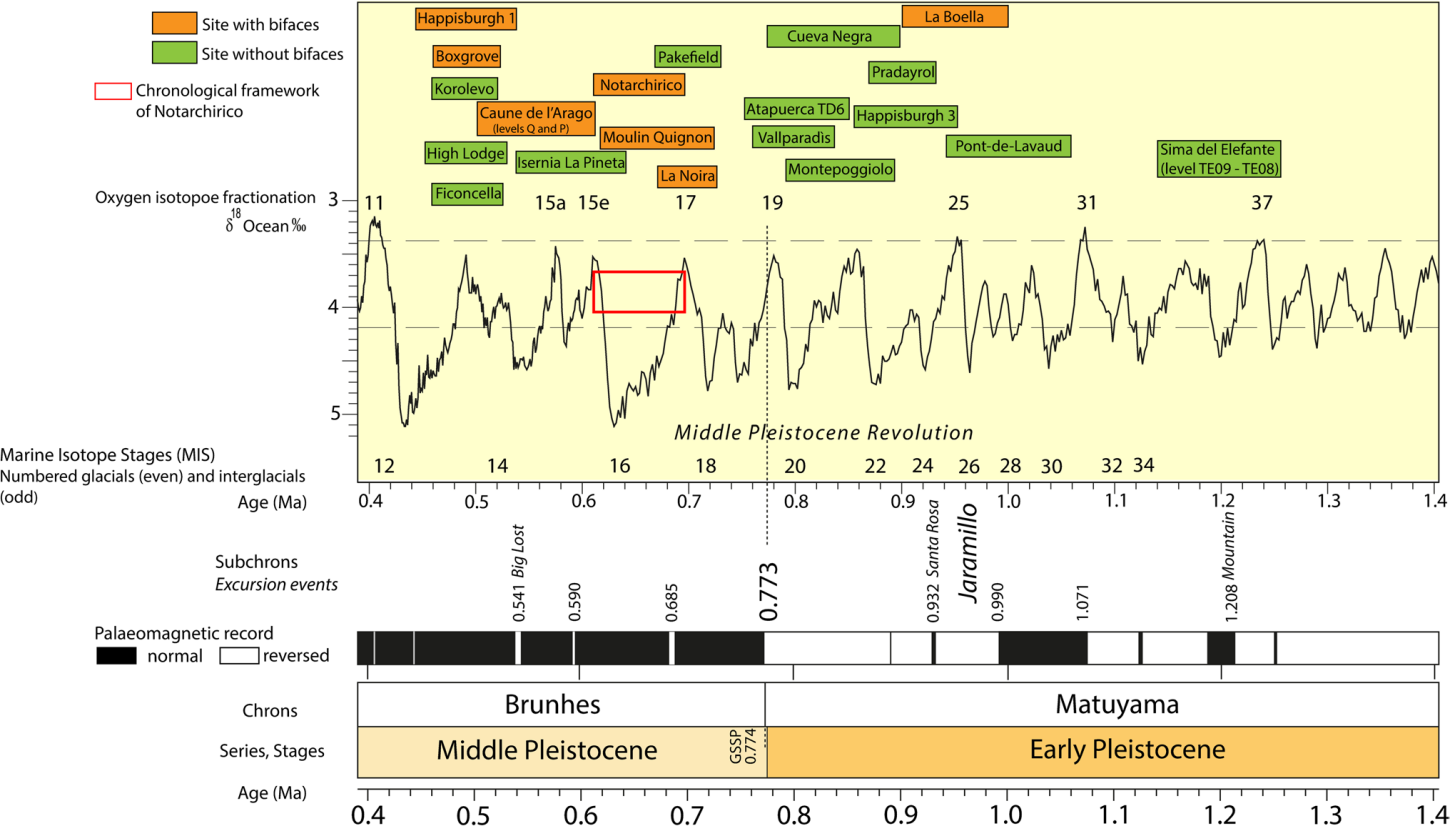
Lower and Middle Pleistocene sites mentioned in the text in relation to chronology, isotopic stages, and palaeomagnetic record. Chronological chart modified from Cohen and Gibbard (2019)

Within the present state of the art, the earliest appearance of large cutting tools (LCTs) and bifaces on the European continent goes back to the Spanish site of Barranc de la Boella (1.0–0.9 Ma), where some crudely made (i.e. presenting a partial or roughly made shaping without management of the bifacial volume) bifacially worked tools were recovered (Mosquera et al., 2016; Vallverdú et al., 2014). The archaeological evidence that characterises the final stages of the Lower Pleistocene (Fig. 1) features the homogeneous presence of core and flake assemblages such as Atapuerca (levels TE08–TE09, 1.2 Mya; Ollé et al., 2013), Barranco Leòn (1.3–1.1 Mya; Agustí et al., 2015), Pont-de-Lavaud (1.0 Ma; Despriée et al., 2018), Happisburgh 3 (900 ka; Parfitt et al., 2010), Monte Poggiolo (850 ka; Peretto et al., 1998), Pradayrol (900 ka; Guadelli, 2012), and Cueva Negra (900–772 ka; Walker et al., 2020), making the findings of la Boella a unique case and raising questions whether a local development of this technology—versus the hypothesis of an African intrusion—might have taken place (Moncel et al., 2015; Mosquera et al., 2013). With the 800 ka threshold approaching (i.e. transition Lower-Middle Pleistocene), major climatic and environmental changes occur at the onset of the Middle Pleistocene (i.e. Middle Pleistocene Revolution), profoundly affecting the peopling of Europe—corresponding to an archaeological hiatus—and triggering the dispersion of new faunal species alongside vegetal turnovers and the diffusion of human groups (*Homo heidelbergensis* and possibly other hominins) from the African and Asian continents (Abbate & Sagri, 2012; Almogi-Labin, 2011; Belmaker, 2009; Blain et al., 2008; Leroy et al., 2011; Manzi, 2004; Manzi et al., 2011; Moncel et al., 2018a; Muttoni et al., 2018). During this time frame, the Acheulean—and bifacial assemblages—show their first diffusion over western Europe (Fig. 1), which also witnesses a global increase in archaeological evidence (Moncel & Ashton, 2018; Moncel et al., 2018b). As a consequence, after a gap of 200 ka from the findings of La Boella, several bifaces and large cutting tools have been found in three key sites: La Noira (700 ka; Moncel et al., 2020a), Notarchirico (Italy, 680 ka; Moncel et al., 2020b), and Moulin Quignon (France, 670 ka; Moncel et al., 2021b). No transition assemblages are recorded during this chronological gap, even though the persistence of core and flake production is reported in contexts such as Atapuerca TD6 (Spain, 800 ka; Ollé et al., 2013; Mosquera et al., 2018; Lombao et al., 2022), Vallparadìs (Martínez et al., 2010; Spain, 800 ka; Garcia et al., 2013b), Pakefield (England, 700 ka; Parfitt et al., 2010), and Isernia La Pineta (Italy, 590 ka; Gallotti & Peretto, 2015), sometimes also attesting the realisation of large-sized tools. The presence of bifaces and other LCTs is not the only innovation among these lithic assemblages, as evidence of more elaborated core technology, frequency of retouched implements, raw material use, and subsistence strategies are generally documented at Atapuerca TD6, La Noira, and Isernia La Pineta (Hardy et al., 2018; Lombao et al., 2019, 2022; Moncel et al., 2021a; Mosquera et al., 2018).

Climatic and environmental changes are essential in triggering human responses, causing abandonments and re-occupations (“back and forth pattern”) of some geographical regions occasionally recorded in the stratigraphic sequences of different sites (Bermúdez de Castro et al., 2013; Davis et al., 2021; Dennell, 2003; MacDonald et al., 2012). During these events, innovative behaviours might have developed as external pressures often foster reactions that can be archaeologically seen and documented (Davis & Ashton, 2019; Key & Ashton, 2022; Moncel et al., 2021a). Therefore, tracking the evolution of these aspects and comparing them with the material culture—alongside the concepts of innovation, persistence, and whether they are detectable from an archaeological perspective—can enable significant insights into the time, place, and modalities of Europe’s colonisation by hominins.

Several works speculated whether these innovations are the outcome of internal behavioural evolution or are due to the arrival of new populations, but undoubtedly, a cognitive shift took place within this chronological framework. According to the available data on the European peopling, the Brunhes-Matuyama shift (780 ka, Lower-Middle Pleistocene boundary) caused a significant break all over the territory, leading to an abandonment of England, France, Spain, and Italy after a moment of continuity between the Jaramillo subchron and MIS 20 (Antoine et al., 2010; Cuenca-Bescós et al., 2015; Davis et al., 2021; Garcia et al., 2013a; Key & Ashton, 2022; Messager et al., 2011; Michel et al., 2017; Muttoni et al., 2010; Preece & Parfitt, 2012). The site of Atapuerca, in level TD6, records an interruption of human occupation right after 800 ka and until 500 ka (Bermúdez de Castro et al., 2013), while other contexts such as Happisburgh 3, Pradayrol, Monte Poggiolo, and Vallparadìs show an absence of human evidence after this threshold. With the onset of interglacial 17 (700 ka) and retreat of the glacial front, a re-occupation, especially at high and middle latitudes (Antoine et al., 2010; Ashton & Lewis, 2012; Preece & Parfitt, 2012), of different areas is witnessed (La Noira stratum a, Moulin Quignon, Pakefield, and Notarchirico)—together with the emergence of bifacial technology—though shortly followed by an abrupt climatic crisis (MIS 16) subsequently causing another abandonment of these regions (Moncel et al., 2021a). The successive interglacials 15 and 13 (the glacial stage 14 is considered to be mild and not so disruptive) are characterised by a prolonged phase of climatic and environmental stasis and, as a consequence, by a reprise in human occupation all over Europe: i.e. Isernia La Pineta, Caune de l’Arago (levels Q and P), Happisburgh 1, Boxgrove, High Lodge, Ficoncella, and Korolevo (Aureli et al., 2016; Barsky, 2013; Falguères et al., 2015; Gallotti & Peretto, 2015; Gibbard et al., 2019; Koulakovska et al., 2010; Roberts & Parfitt, 1999; Zanazzi et al., 2022).

The related lithic assemblages show a mixture of handaxes, diverse LCTs, pebble tools, and core and flake production, attesting to a diversified range of subsistence strategies and functions of the sites (García-Medrano et al., 2019; Moncel et al., 2018c; Muttillo et al., 2021). Nonetheless, the reason for the absence of bifacial tools in some of these contexts is still a debated topic. Aside from more common issues, such as the quality and morphology of the available raw materials that could prevent the realisation of handaxes, recent works highlighted that there is much more than “the traditional concept of the biface” (Moncel et al., 2015, p. 305) to what we define as Acheulean and, more in general, to what is perceived as a sign of complexity (Moncel & Ashton, 2018). The ability to realise dimensionally large implements, the presence of structured centripetal or discoidal cores—implying *debitage* conducted regardless of the original shape with the possibility of subordinating the morphological criteria to the production goals—the degree of retouch on flakes, and the flexibility itself in the concept of *façonnage* and *debitage* are among the addressed matters for the contextualisation of the “European Acheulean” (Martínez & Garcia Garriga, 2016; Moncel, 2017; Rocca et al., 2016).

In this geographical and chrono-cultural framework fits the site of Notarchirico, being the only one recording a continuous human occupation during stages 17 and 16 across the whole sequence (Moncel et al., 2020b; Pereira et al., 2015) and attesting to one of the earliest evidence of bifaces together with core and flake production. The prolonged and repeated frequentation of the site during glacial phases might be due to its southern geographical position, acting as a sheltered area for human groups during the major climatic crisis and as a possible starting point for re-peopling Europe during the earliest phases of the Middle Pleistocene. Thus, the analysis of the lithic production of Notarchirico throughout the entire stratigraphic sequence may offer several hints about the notions of continuity and innovation within European Lower Palaeolithic lithic assemblages, not to mention the opportunity of reconstructing hominin subsistence strategies and their evolution over a significant climatic and chronological range.

In this work, we focused our research on the core, flake, and tool production of the lowermost portion of the sequence of Notarchirico (layers F to I2) chronologically framed between 695 and 670 ka—thus penecontemporaneous to the sites of Moulin Quignon and La Noira—which proved to be a crucial moment for western Europe.

## Notarchirico

**Fig. 2:**
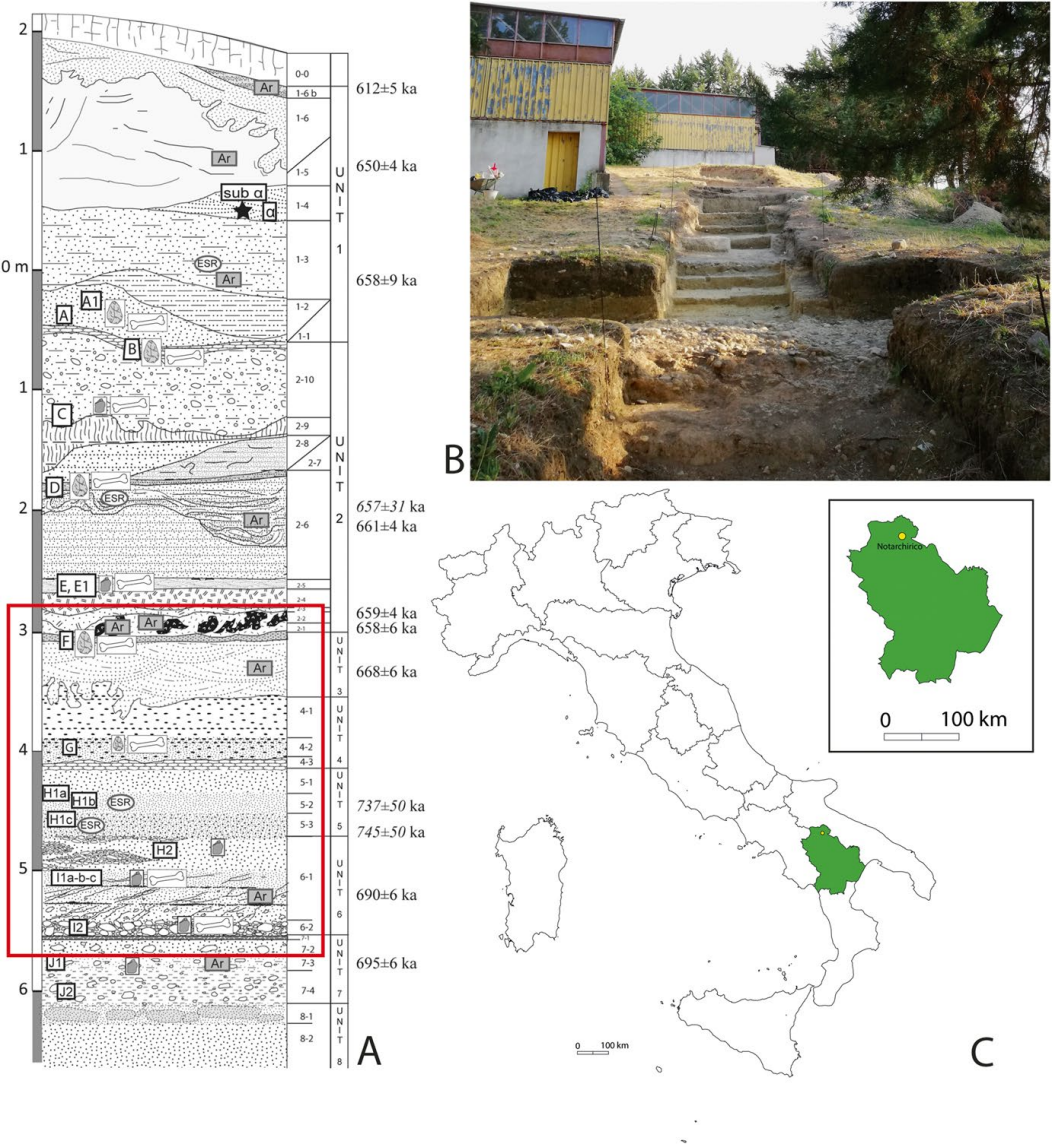
**A** Complete stratigraphic sequence of Notarchirico. Dates in italics by ESR-U-Th. Other dates by 40Ar/39Ar. Legend is available in Moncel et al. (2020b). The red square indicates the archaeological layers analysed in this work. **B** Photograph of the new excavations on the Notarchirico hill (on the left is the M. Piperno’s fieldwork building). **C** Location of the site

The site of Notarchirico lies within the fluvial-lacustrine basin of Venosa (Piano Region sedimentary formation), a few kilometres outside of the village of Venosa (PZ, Basilicata) in southeastern Italy (Fig. 2). It is an open-air site originally discovered by M. Piperno in 1979 and extensively excavated for more than 30 years on an area of approximately 133 m^2^.

A 7-m-thick sequence of fluvial sediments was unearthed, including 11 archaeological layers, five of which contained bifaces (Fig. 2A) (Piperno, 1999). The sequence is also rich in volcanic material due to the eruptive activity of the Vulture stratovolcano, located 10 km from the site (Lefèvre et al., 2010). The archaeological material consisting of lithic implements associated with large herbivore carcasses lies on beds of pebbles and cobbles corresponding to shallow paleochannels and lakeshore remains. Recent geochronological dating (ESR, 40Ar/39Ar) established the chronological limits of the sequence excavated by Piperno between 675 ka (layer F) and 610 (layer α), corresponding to the glacial stage 16 (Pereira et al., 2015). A fragment of a hominin femur was found in the upper part of the deposit (layer α) and attributed to *Homo heidelbergensis* (Belli et al., 1991), making it the oldest human fossil in the Italian Peninsula.

The faunal assemblage, described by Cassoli et al. (1999), is attributed to the Isernia faunal unit and is mainly composed of the straight-tusked elephant (*Palaeoloxodon antiquus*), fallow deer (*Dama clactoniana*), and two species of bovids (*Bos primigenius* and *Bison schoetensacki*). The presence of other herbivores, such as *Megaloceros solilacus* and *Cervus elaphus*, is attested to in the higher levels (α, sub-α, A/A1, B, and D), while the absence of carnivores along the whole sequence was reported. The micromammals, analysed by Sala (1999), consist of *Pliomys episcopalis*, *Chionomys nivalis*, *Microtus* sp., and *Arvicola cantiana*, suggesting a cold climate typical of a cold environment during a glacial stage. The palynological results, conducted by Cattani in 1996 only at the top of the deposit, show an open and cold environment—in agreement with the dates (MIS 16)—consisting of Poaceae meadows with limited presence of trees (*Pinus sylvestris*, *Quercus pubescens*, *Quercus ilex*, *Corylus*, *Carpinus*, *Fraxinus*, and *Ulmus*).

The lithic industry is realised on local raw materials, including chert and limestone pebbles and nodules, collected in secondary positions along the river banks/lakeshores (Moncel et al., 2019; Piperno, 1999; Santagata et al., 2020). Both heavy-duty tools and core and flake production are attested. The heavy-duty components are on limestone and chert, consisting of various unifacial and bifacial pebble tools, cleavers, pointed elements, and bifaces. Though a low standardisation was observed for this production, the recent revision of the bifaces and LCT demonstrated their affinities with the Acheulean cultural techno-complex s.l. (Moncel et al., 2019; Santagata, 2016; Santagata et al., 2020).

Chert nodules and pebbles of small dimensions are used to produce small flakes (15–20 mm) through unifacial/multifacial *debitage*. Larger flakes (50–100 mm) are rarer and mainly obtained from limestone or chert. Some cores showed alternate *debitage* resembling a discoid conception but no platform preparation. Retouched tools are also attested to, consisting of scrapers, notches, and denticulates. Freehand and bipolar on anvil percussion are both attested for the *debitage*.

Since 2016, new investigations have been taking place at Notarchirico to explore the bottom of the sequence excavated by Piperno below layer F (Moncel et al., 2020b). A 30-m-long trench was thus opened on the side of Notarchirico hill, covering a surface of 8 to 26 m^2^. The excavations led to the identification of five lithostratigraphic units (3 to 8), including five new archaeosurfaces (G, H, I1, I2, and J) in addition to the previously known layers G and F (Fig. 2). All of these, except for layer J, bear evidence of human frequentation with layers F, G, I1, and I2 providing evidence of recurrent occupation. On the other hand, layer H is thought to record a sporadic/short-term site frequentation phase.

The basal lithostratigraphic units of the deposit of Notarchirico (units 8 to 6) exhibit low-energy fluvial sedimentation along regular inputs of volcanic materials, while in the upper units (5 to 3), the sedimentation displays higher energy currents and mainly volcano-derived remains. The archaeological horizons of the bottom of the sequence are associated with the lithostratigraphic unit 3 (layer F), the bottom of sub-unit 4.2 (layer G), the bottom of sub-unit 5.3 (layer H), sub-unit 6.1 (layer I1), sub-unit 6.2 (layer I2), and sub-unit 7.4 (layer J) (Fig. 2). According to the available lithostratigraphic analysis (for a more detailed description, see J-P. Raynal, P. Dugas, G. Jouanic and A. Queffelec in Moncel et al., 2020b), the different facies identified corresponds to fills of meandering paleo-channels, crossed in places by the action of low energy currents. The finer component of the deposits derives from the alteration of volcanic fallout, which is particularly common between units three and four. The presence of cobbles and gravels incorporated in the layers corresponds to slope destabilisation processes intervening after the arrival of masses of tephra and the release of lateral contributions from older conglomeratic deposits.

From top to bottom of the stratigraphic sequence, layer F is described as a bed of cobble-pebbles cross-bedded with volcano-derived and non-volcanic sands with a thickness of approximately 20 cm overlying another 20-cm-thick layer of black volcanic sands. Dark-grey volcanic sands characterise layer G (lithostratigraphic unit 4.2, 30 cm thick) dispersed over a coarse sandy sub-unit (lithostratigraphic unit 4.3) with cobbles and sub-angulus gravels (30 cm thick). Layer H (30 cm thick) features a silty-sandy deposit with a few micro-beds of dark minerals. Local lenses of small pebbles characterise layer I1 in the first 15–30 cm, while coarse sands and beds of more or less dense gravels with millimetric anastomosed crusts are distributed in the remaining 45 cm of the layer’s thickness. Layer I2 presents a similar characterisation to I1 though displaying a denser accumulation of cobbles and smaller elements with limestone pebbles and a few fine-grained sandstone cobbles and flint nodules over a 10–15 cm thickness. Cobbles in a clayish volcano-derived matrix of 30 cm of thickness underlying a 10-cm-thick tephra-derived coarse with some cobbles characterise layer J.

Datings using 40Ar/39Ar and ESR methodologies placed the chronology of the new sequence between 695 and 670 ka in correspondence with the end of the interglacial 17 and the beginning of glacial 16, providing evidence for continuity in the human occupation of the site (Moncel et al., 2020b). As in the upper part of the sequence, the archaeological material of lithic artefacts associated with faunal remains lies within beds of pebbles and cobbles of approximately 10–30-cm thickness, related to paleo water channels and lakeshores. Layers F and I2 show a dense bed of pebbles in situ, while layers G and I1 are more disturbed.

The new palaeontological analysis available for layers F, G, I1, and I2 (for a more detailed description of the faunal remains recovered from the new excavations, see B. Mecozzi, A. Iannucci and R. Sardella in Moncel et al., 2020b and related supplementary material) (Table 1) highlighted the presence of *Palaeoloxodon antiquus* along the whole sequence, followed by cervids (*Praemegaceros* sp., *Dama* cf. *clactoniana*, and *Cervus elaphus*) and bovids (bison and aurochs) while no carnivores have been found so far. Two new species were reported: *Hippopotamus antiquus* (layers G and I1) and *Macaca sylvanus* spp. (layer G; Mecozzi et al., 2021). Overall, the faunal assemblage of these layers matches the attribution to the Isernia faunal unit made for the upper part of the sequence. Concerning micromammals, few remains were recovered, mainly from layer I1: *Arvicola mosbachensis*, *Microtus* (*Terricola*) cf. *M.* (*T.*) *arvalidens*, and *Microtus* cf. *M. nivaloides* were identified, with the *A. mosbachensis* being one of its earliest occurrences (Moncel et al., 2020b). The attribution to the beginning of Early Toringian (*Arvicola-Microtus* zone, *Arvicola mosbachensis* subzone 3) is in accordance with the data from previous excavations.

**Table 1:** Mammal species from layer F-I2 (modified after Moncel et al., 2020b Supplementary Material)

Layer	F	G	I1	I2
Species				
*Palaeoloxodon antiquus*	X	X	X	X
*Hippopotamus antiquus*		X	X	
*Bison schoetensacki*	X	X	X	X
Bovidae indet	X		X	X
*Praemegaceros solilhacus*			X	X
*Cervus elaphus*			X	X
*Macaca sylvanus* spp.		X		

The archaeozoological analysis did not point out cut marks or carnivore tooth marks due to the dire state of preservation of the bone surfaces and the high fragmentation rate. Most of the modifications detected on the bones, such as abrasion, corrosion, and concretions, may be related to the effects of hydraulic transportation and trampling (abrasion) and exposure to water (corrosion and concretions). The abundance of short elements for all species and the amount of post-depositional dry bone fractures confirm the presence of a lacustrine environment and a secondary origin of the deposit (for a more detailed description, see C. Daujeard and A. Curci in Moncel et al., 2020b). Seemingly, the animals died naturally near these lakeshores/water channels and were secondarily transported and accumulated. Therefore, an anthropic origin for the bone accumulation, perhaps with carnivore contribution, cannot be fully supported even though the interaction between hominins and animal carcasses has been assessed based on lithic use-wear (Moncel et al., 2020b).

The lithic industry from these layers consists of more than 900 artefacts realised on chert nodules and various silicified limestone pebbles locally collected in a secondary position. The artefacts can be divided into two main groups: core and flake (analysed in this work) and heavy-duty components (Table 2). The goal of the *debitage* production is mainly small-sized flakes (10–20 mm) and, more rarely, larger flakes (40–120 mm) employing different types of knapping strategies (discoid, unifacial, multifacial, centripetal, etc.). Retouched tools are also present (denticulates, scrapers, and pointed tools). In addition, hominins selected small chert nodules (20–40 mm) to shape through an abrupt or denticulate retouch. Various artefacts characterise the heavy-duty component with a low morphological standardisation (unifacial, bifacial, and trifacial pebble tools; diverse LCTs; rabots; and chopping tools). These are mainly obtained from limestone pebbles, with only one chert implement. The bifaces, on the other hand, show complete control of the bifacial and bilateral symmetry. They are realised mainly on limestone and the few locally available chert pebbles. The shaping process covers a large portion of the periphery and surface of these tools by one or several series of removals with evidence of retouch to regularise the cutting edges. The cross-sections are symmetrical or plano-convex, often presenting a cortical base. The recent analysis also highlighted evidence of recycling on the cutting edges of one of the handaxes. A total of six bifaces were recovered from layers F and G, further postponing the rise of the Acheulean cultural complex in this region (for a detailed description of the heavy-duty component from layers F-I2 of Notarchirico see Moncel et al., 2020b).

**Table 2:** Heavy-duty component from layers F-I2 (Moncel et al., 2020b)

Layer	F	G	H	I1	I2
Unifacial convergent LCT tools	5	6			2
Bifaces	4	2			
Unifacial pebble tools	34	15	1	9	5
Bifacial pebble tools	6	2		2	1
Pointed unifacial pebble tools	10	6		2	
Pointed bifacial pebble tools/LCTs	4			1	
Trifacial pebble tools		1		1	
Rabots on pebbles	5	2			1
Quadrangular unifacial tools	2				
Broken pebbles with impacts + isolated removals	52	31		1	

Preliminary use-wear and residue analyses (see C. Lemorini and B. Hardy in Moncel et al., 2020b) have been performed on flakes and tools. The analysis revealed the presence of different post-depositional processes on the artefact surface: patina, gloss (a consequence of the mechanical action of the water flow), striations, and mechanical alterations. Despite these processes, it was possible to observe the presence of use-wear. The results highlighted the interaction with soft to hard materials (fleshy tissue and woods have been identified so far), mainly worked by cutting and scraping and, to a lesser extent, by mixed actions like engraving. Seemingly, the *debitage* implements were employed for different activities and purposes, not only related to food processing (Moncel et al., 2020b).

## Materials and Methods

This work focuses on the most significant quantity of the lithic assemblage of Notarchirico: core and flake production and retouched nodules from archaeological layers F, G, H, I1, and I2, belonging to the new fieldwork started in 2016. All the lithic pieces of this classification (i.e. cores, flakes, retouched flakes, and retouched nodules) recovered from these layers were analysed and studied. Layer F was excavated over 10 m^2^, layer G over 11 m^2^, layer H over 8 m^2^, layer I1 over 14 m^2^, and layer I2 over 20 m^2^. The lithic material from layer J, consisting of a few artefacts, is probably not in situ and has been removed from this analysis (Moncel et al., 2020b). This material was selected because of the great diffusion of small-sized flake assemblages within the Italian Peninsula during the Middle Pleistocene, and, unlike bifacial and large cutting tools of Acheulean affiliation, they are an emblematic trait of this chrono-cultural framework that still needs to be properly contextualised.

The technological analysis (Inizan et al., 1999) and the concept of *chaîne opératoire* (Boëda et al., 1990; Geneste, 1991; Leroi-Gourhan, 1965; Roche, 2005) have been applied to study the lithic material to conceive all the phases of the flaking activity as a single process from the raw material selection through the obtainment of flakes until their abandonment. The hierarchy of flaking surfaces, removals organisation, and size were considered on cores to evaluate the knapping strategies employed by the hominins and their degree of complexity (Moncel et al., 2020a). The use of terms like unifacial, bifacial, and multifacial applied to cores is meant to describe the number of the knapping surfaces, while “unipolar”, “convergent”, “crossed”, “orthogonal”, “centripetal”, and “bipolar” were applied to describe the distribution and the organisation of the removals over the knapping surfaces. Since bifacial and multifacial cores of Notarchirico are the outcome of multiple separate unipolar knapping events due to core rotation rather than a surface hierarchisation, the description of the removal organisation for these latter categories was removed in favour of terms like SSDA (*systeme par surface de débitage* alterné) (Ashton et al., 1992; Forestier, 1993) that better describe these type of knapping strategies.

For flakes, the presence and position of the cortex, butt characteristics, removals organisation, the incidence of backed margins, and, when present, the location, delineation, and angle of retouch were recorded. The description of retouched nodules was made using the same criteria applied to retouched flakes for a proper comparison.

Concerning the typological classification employed to classify the retouched component (flakes and nodules), despite the evident limitations that such an approach implies through the creation of artificial categories (especially when dealing with such old archaeological palimpsests), we decided to use a basic typological description of these tools to facilitate the comparison from a technological point of view with other lithic assemblages where a similar approach was applied. We want to underline that the adoption of terms like denticulate, scraper, and notch is made only to describe the morphological organisation of the retouch on the lithic pieces without inferring the functional implications of these lithic artefacts. For instance, we consider “scraper” the presence of regular edge modifications (i.e. retouch) on a cutting edge regardless of its length, while “denticulate/notch” results in a non-linear configuration of the retouch. “Point” and “beak” describe retouch to configure a pointed shape/termination of the lithic object, while the term “composite tool” was applied to describe a mixture of these characteristics on the same artefact.

The raw material identification was made according to the petrographic and chemical analyses performed by Eramo et al. (in Moncel et al., 2020b), where four main lithotypes of chert were identified: silicified litharenites, nodular chert, vitreous chert, and radiolarite. The presence of limestone is reported as well. Such lithotypes occur in the polygenic pebbles, and cobbles lags formed in the fluvial-lacustrine environment of the area of Notarchirico (Synthem of Palazzo San Gervasio; ISPRA, in press) as products of the erosion of the outer geological units of the southern Apennine formed after the evolution from late Triassic to Miocene of a deep-sea basin on passive margin (Lagronegro basin) to a foredeep basin (Irpinian basin) characterised by flyschoid sequences (Pescatore et al., 1999).

To bring order to the terminology used to classify lithotypes in previous studies and the present work, the term *chert* is intended here as a generic group used for fine-grained siliceous sedimentary rocks following Tucker (2001). Usually, in the geological record, cherty rocks are subdivided into bedded types resulting from primary accumulation (e.g. radiolarites and diatomites) and the nodular type of diagenetic origin (Greensmith, 2012; Trewin & Fayers, 2005). Excluding radiolarites, although the other identified chert types can be traced to facies and diagenetic conditions of turbiditic systems, the term *flysch chert* refers to silicified litharenites (Eramo et al., in preparation).

## Results

A total of 591 pieces from layers F, G, H, I1, and I2, which will be discussed separately, were analysed and studied (Table 1). The lithic assemblage is mainly composed of flakes and flake tools, followed by retouched nodules and cores in a minor percentage (Table 3). Small retouched nodules constitute a peculiar aspect for this site, representing 20% of all the analysed pieces and being more or less constant along the entire stratigraphic sequence (Table 3). The *debitage* production was achieved through a direct percussion by hard hammer technique. Nonetheless, the use of anvil percussion (both for *debitage* and retouch actions) cannot be entirely ruled out, given the importance of this technique in similar contexts exploiting small-sized raw materials (Isernia La Pineta) and its well-known difficulties in being adequately distinguished from direct percussion (Pargeter & Eren, 2017; Peña, 2015; Peretto, 1994; Sánchez-Yustos et al., 2017; Vergès & Ollé, 2011).

**Table 3:** List of analysed lithic material

Layers	F		G		H		I1		I2		Total	
Categories	*n*	%	*n*	%	*n*	%	*n*	%	*n*	%	*n*	%
Cores	8	5.4	24	11.8	0	-	22	12.8	9	23.1	63	10.6
Flakes (unretouched)	104	70.3	58	28.4	15	53.6	77	44.8	20	51.3	274	46.4
Flakes (retouched)	30	20.3	52	25.5	4	14.3	34	19.7	4	10.2	124	21.0
Retouched nodules	6	4.0	70	34.3	9	32.1	39	22.6	6	15.4	130	22.0
Total	148	-	204	-	28	-	172	-	39	-	591	-

The global distribution of the raw materials in the analysed sample highlights the predominance of chert lithotypes (Table 4), with flysch chert being the most represented in all the technological categories (87%), followed by nodular chert (10%) and radiolarite (3%). As previously mentioned, hominins collected chert nodules in a secondary position. For this reason, the percentage of cortical patches on the support is relatively low (rolling, breakages, fragmentation, etc.). The development of the neocortex on the nodules is recorded alongside the massive presence of natural surfaces (i.e. surfaces naturally deprived of the cortex and without a neocortex formation).

**Table 4:** Distribution of the lithotypes

Layers	F	G	H	I1	I2	Total	
Lithotypes and technological categories						*n*	%
Flysch chert							
Cores	7	17	-	18	8	50	8.9%
Flakes	89	48	11	67	19	234	41.9%
Retouched flakes	25	40	3	28	4	100	17.9%
Retouched nodules	5	61	8	26	3	103	18.4%
Total						487	87.1%
Nodular chert							
Cores	1	1	-	2	-	4	0.7%
F lakes	7	8	4	8	-	27	4.8%
Retouched flakes	3	7	-	1	-	11	2.0%
Retouched nodules	1	6	1	5	2	15	2.7%
Total						57	10.2%
Radiolarite							
Cores	-	1	-	-	-	1	0.2%
Flakes	1	1	-	-	-	2	0.4%
Retouched flakes	-	1	-	2	-	3	0.5%
Retouched nodules	-	3	-	5	1	9	1.6%
Total						15	2.7%
Total						559	-

The state of preservation of the lithic material can be globally considered as ranging between good and medium, with evidence of poorly preserved and “fresh” artefacts. Most of the sample shows different degrees of patination and superficial alteration that prevented the correct assessment of each lithotype’s colour but did not influence the technological analysis. Evidence for roundings of the edges or natural removals on the pieces is relatively common though the presence of fresh cutting edges on the artefacts is also frequent. Thus, the lithic pieces were accurately selected, discarding from the analysis all the artefacts not presenting clear knapping marks or clear removal organisation.

### Layer F

**Fig. 3:**
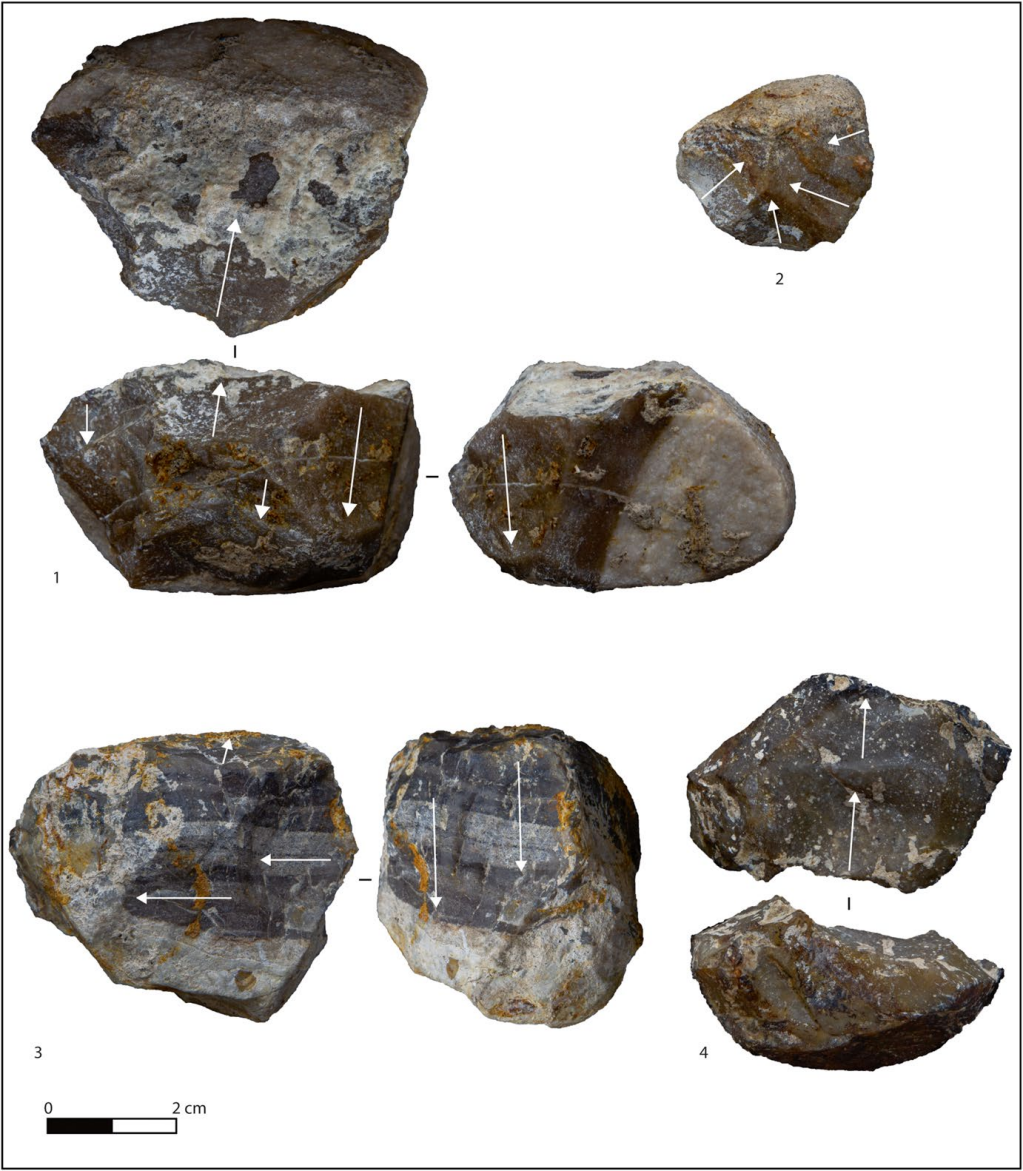
Cores from layer F. 1 Multifacial core on round nodule of flysch chert. 2 Unifacial centripetal core on small nodule of flysch chert. 3 Multifacial core on flysch chert. 4 Unifacial unipolar core on flysch chert

**Fig. 4:**
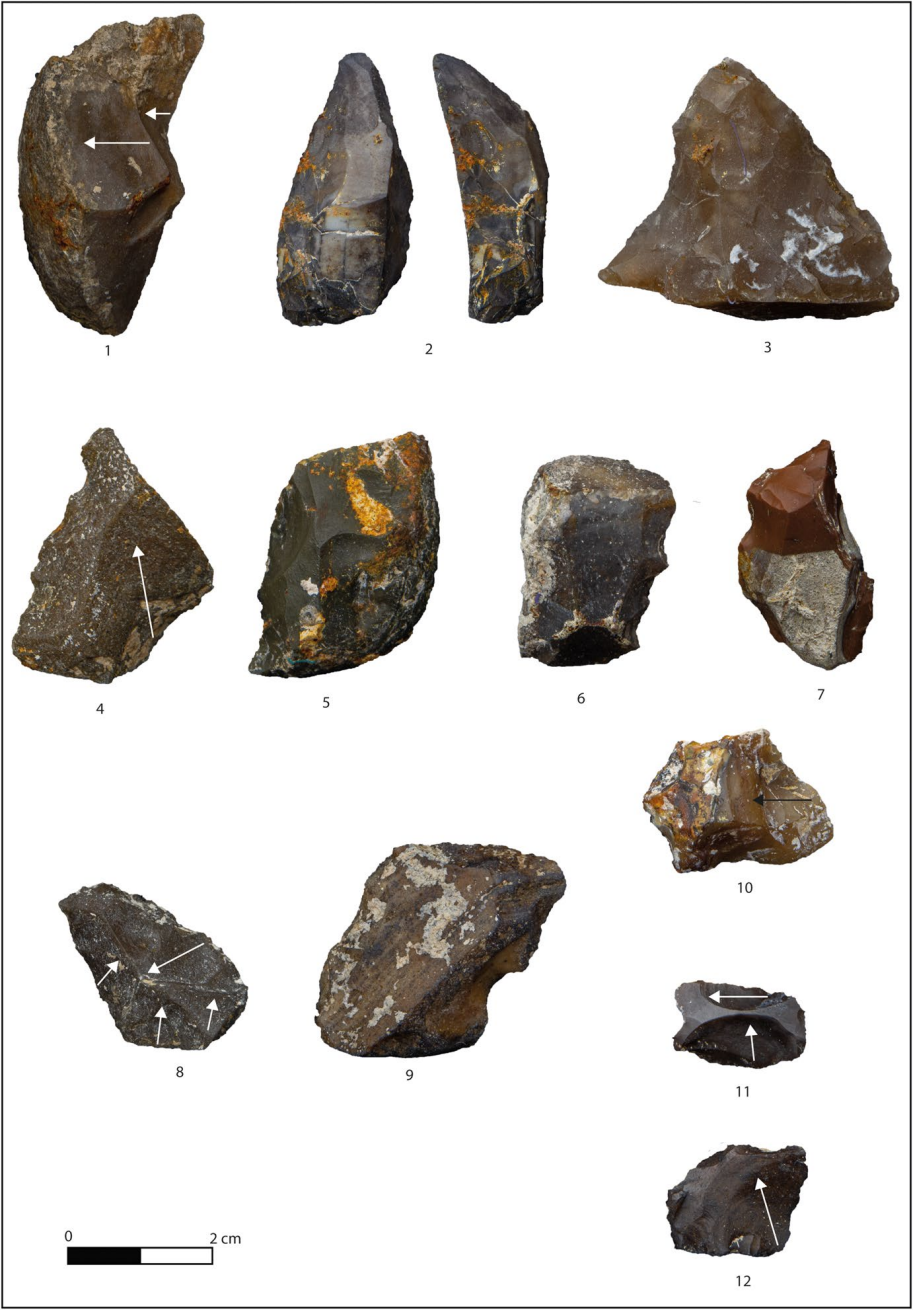
Layer F: debitage products and nodules. **1** Débordant flake on flysch. **2** Scraper on flake of nodular chert. **3** Convergent scraper/pointed tool on flake of nodular chert. **4** Denticulate on flysch flake. **5** Scraper on flysch flake. **6** Denticulate on débordant flysch flake. **7** Convergent scraper/pointed tool on débordant flake of nodular chert. **8** Centripetal flake on flysch. **9** Notch on nodule of flysch. **10**–**11** Flake with orthogonal removals on flysch. **12** Flake on nodular chert

There are 148 lithic implements from layer F (Table 3; Figs. 3 and 4). Flakes and tools represent 90% of the whole layer, with rare cores and retouched nodules, and flysch being the most exploited raw material (Table 4). The privileged support for cores is small, cubic, or roundly shaped nodules (20–80 mm; see Table 5) bearing cortex on one or two faces (Fig. 3). Cores are equally knapped on one or more knapping surfaces according to the available natural convexities, showing mainly unipolar removals followed by centripetal and bipolar ones (Fig. 3; Table 6), with a mean of three removals per core. The *debitage* often uses cores edges as a technical expedient to speed up production, explaining the high ratio of backed flakes. Multifacial cores present alternate flaking recalling an SSDA type (*systeme par surface de débitage*; Ashton et al., 1992; Forestier, 1993), but there is also evidence of a small core selected for just two removals (Fig. 3, n 4). Striking platforms are natural in most cases (7 out of 11), but a preparation of the surfaces is attested nonetheless.

**Table 5:** Dimensional values

Layers	F			G			H			I1			I2		
	l	w	t	l	w	t	l	w	t	l	w	t	l	w	t
Flakes	*n* = 75			*n* = 40			*n* = 9			*n* = 57			*n* = 19		
Min	12.5	9.1	3.2	6.4	9.2	2.1	8.9	9.3	3.5	5.3	7.4	1.9	14.1	12.4	5.5
Max	59.9	56.5	22.3	62.3	49.5	18.2	23.2	16.9	6.9	45.6	33.7	21.7	37.2	32.4	18.0
Mean	26.2	22.4	9.4	21.4	20.2	8.21	14.0	13.4	5.2	19.9	18.5	8.4	23.9	21.0	10.3
Tools	*n* = 17			*n* = 41			*n* = 2			*n* = 22			*n* = 4		
Min	22.5	14.7	6.6	13.7	12	4.6	11.4	13.7	4.6	9.3	10.9	4.2	18.4	16.9	7.3
Max	61.6	41.2	22.4	68.1	47.5	25.5	15.3	15.7	4.9	48.8	37.5	19.2	60.1	31.4	17.6
Mean	34.7	25	12.4	26.8	22.3	11.8	13.3	14.7	4.7	26.7	23.2	10.3	38.2	24.5	12.6
Nodules	*n* = 6			*n* = 67			*n* = 9			*n* = 38			*n* = 5		
Min	18.2	17.3	6.65	11.5	12.1	5	12.8	11.5	5.1	9.8	11.1	4	15.2	12.1	3.9
Max	73.2	53.7	30.1	53.1	38.7	24.0	34.4	29.5	13.5	42.5	36.3	21.2	31.9	25.1	13.2
Mean	32.0	25.8	13.0	26.0	20.7	12.7	21.9	19.6	9.3	24.9	20.0	11.9	21.0	18.7	8.9
Cores	*n* = 6			*n* = 19			*n* = 0			*n* = 16			*n* = 9		
Min	26.1	30.7	17.1	18.9	20.2	15.4	-	-	-	19.7	18.1	11.7	18.7	19.5	15.5
Max	77.4	59.4	51.3	98.9	87.7	67.3	-	-	-	72.7	47.3	29.9	82.9	70.2	55.2
Mean	45.4	43.1	34.2	41.8	36.3	28.2	-	-	-	35.0	28.9	20.1	46.2	37.7	27.6

**Table 6:** Core classification

Layers	F	G	H	I1	I2	Total
Core classification						
Unifacial						
Unipolar	2	11	-	9	4	26
Convergent	-	-	-	-	1	1
Bipolar	-	1	-	-	-	1
Centripetal	1	-	-	3	1	5
Total						33
Bifacial	2	9	-	8	1	20
Multifacial	2	3	-	-	2	7
Total	7	24	-	20	9	60

Flakes (*N* = 104) and tools (*N* = 30) exhibit a small quadrangular shape slightly longer than wide, with the latter being bigger and longer than the former (Table 5). The presence of residual cortex is low (19%) and usually located on the lateral margins of the supports (Fig. 4). The removal analysis highlights a mixture of knapping strategies: unipolar and convergent scars are the most common (41%), followed by orthogonal (17%), centripetal (10%), crossed (4.5%), and bipolar (4.5%) with 8% of undetermined. The incidence of natural-backed margins is high for flakes and tools (49%), and they are frequently opposed to cutting edges or retouched ones (see Fig. 4, n 1, 2, 5, and 6). The striking platforms are predominantly flat (49%), while the surface preparation is rare and attested by a few dihedral (9%) and facetted (2%) butts, primarily associated with orthogonal and centripetal removals. On the other hand, the exploitation of natural (13%) and cortical (7%) platforms is more frequent. The presence of punctiform and linear butts is due to the high number of small-sized flakes. The angle flaking has a mean value of 105°, regardless of the platform type, showing a homogeneous use of surfaces.

Retouch is located on the dorsal face for most cases (27 out of 30 pieces), and it seems to affect, to the same degree, single sections of the flakes’ margin, various portions or the entire perimeter (Table 7). The extension of the retouch is equally marginal, abrupt, or invasive, sometimes even combined, regardless of the flake type and, more rarely, characterised by a single removal (Table 5). The identified tools (Table 8) are mainly denticulates, scrapers retouched on one edge, followed by some notches, points (beaks and more or less convergent pointed retouched edges), and composite tools (Fig. 4, n 2–7).

**Table 7:** Characterisation of the retouch (position and extension) of retouched flakes (F.) and nodules (N.)

Layers	F		G		H		I1		I2		Total	
Retouched tools	F	N	F	N	F	N	F	N	F	N	F	N
Position												
Direct	27	4	38	51	4	6	25	32	4	6	98	99
Inverse	1		6	10	1	1	4	2			12	13
Direct and inverse	2	2	8	6		1	5	5			15	14
Extension												
Abrupt	7	4	23	40	1	4	12	22	1	4	44	74
Abrupt and invasive	2	1	4				3	1			9	2
Covering	2		1	1							3	1
Invasive	7		6	2				2			13	4
Marginal	6	1	10	7	3		12	3	2	1	33	12
Marginal and abrupt	1		3	2			2	1			6	3
Marginal and invasive	2		2				1				5	
Single	3		3	15	1	4	4	10	1		12	29

**Table 8:** Typology of retouched flakes (F.) and nodules (N.)

Layers	F		G		H		I1		I2		Total	
Type	F	N	F	N	F	N	F	N	F	N	F	N
Beak			1	7	1			2		1	2	10
Beak and denticulate							1				1	
Denticulate simple	9	2	5	13			5	9	1		20	24
Denticulate double		1	2					1			2	2
Denticulate convergent			4	1		1	3		1		8	2
Notch (single)	2		5	15		4	6	9	1	1	14	29
Notch and scraper			2	1			1	1			3	2
Point	2		3	2			2	1			7	3
Point and scraper	1		1								2	
Scraper simple	10	3	19	19	3	3	9		1	3	42	28
Scraper double	1		5	4			1	2			7	6
Scraper convergent	3			5			1	11		1	4	17
Total	28	6	47	67	4	8	29	36	4	6	112	123

Significantly, few retouched nodules were recovered from this layer (*N* = 6; see Fig. 4, n 9). They are morphologically and dimensionally similar to retouched flakes (Table 5). The retouch is unifacial in four cases, bifacial in two, usually abrupt and located on two adjacent margins of the support (Table 7). Typologically, there are scrapers and denticulates (Table 8).

### Layer G

Layer G is the richest level of Notarchirico. The distribution of lithotypes always reveals a predominance of flysch chert, but there is a slight increase in nodular chert and radiolarite (Table 4). Retouched nodules (*N* = 70) are the most common artefacts, followed by flakes (*N* = 58), retouched flakes (*N* = 52; showing a higher ration compared to the other layers), and, lastly, cores (*N* = 24).

**Fig. 5:**
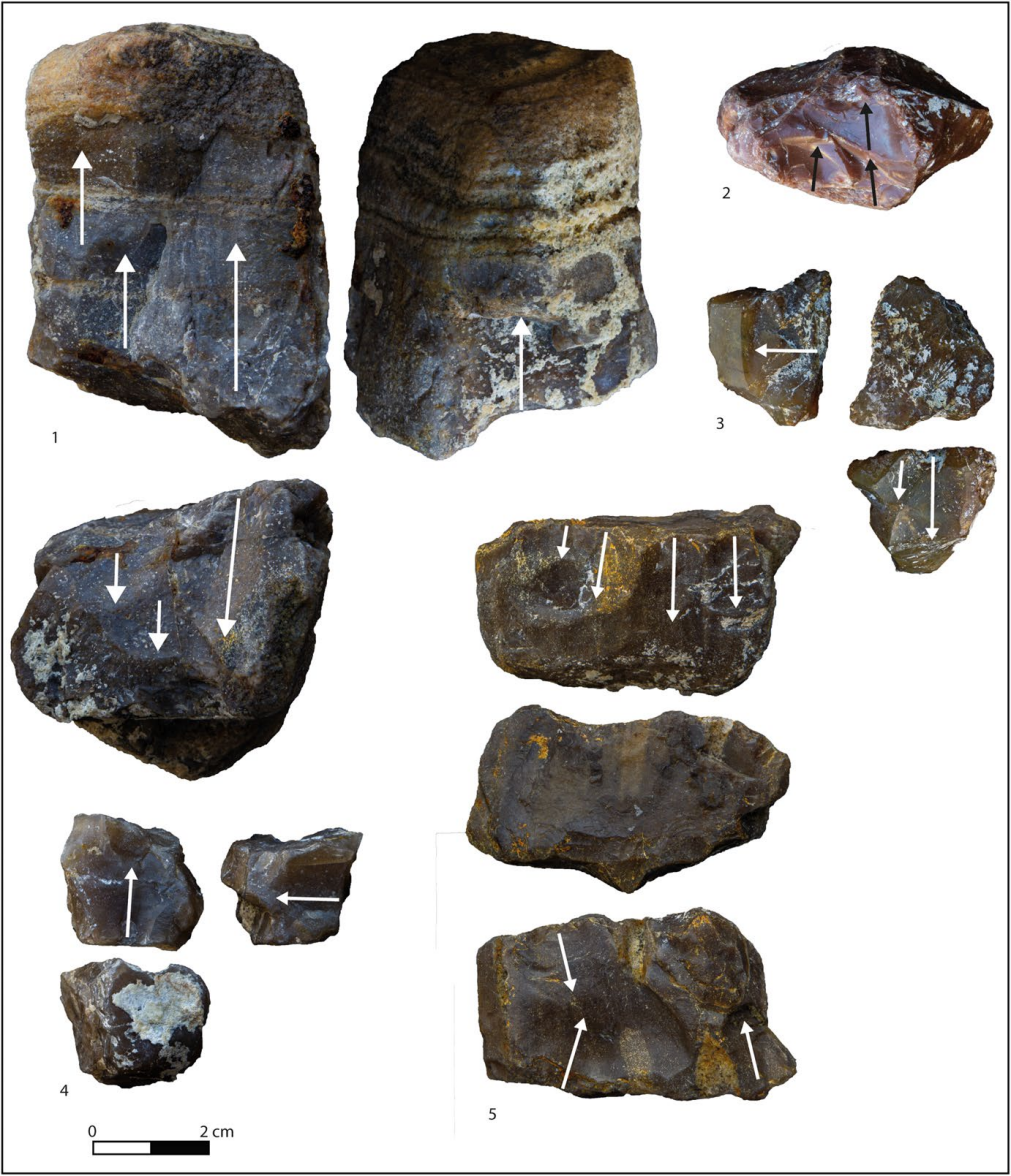
Layer G: cores. 1 Multifacial core on flysch. 2 Unifacial core on radiolarite. 3 Semitournant core on flysch chert. 4 Multifacial core on flysch. 5 Multifacial core on flysch

Cores, realised on cubic or rounder nodules, show a complete absence of cortex (*N* = 16) or a portion on one face (*N* = 8) due to the secondary origin of the nodules’ deposit and are characterised by a lower quality of the raw material (Fig. 5). They are mainly exploited on one or two knapping surfaces with unipolar removals (a mean value of 3 per core), occasionally producing semitournant supports (Table 6; Fig. 5, n 3). Among bifacial cores, there is a fragmented one with alternate flaking exploited through a peripherical striking platform (resembling a discoid conception). Multifacial cores display an SSDA (*systeme par surface de débitage* alterné) conception being the outcome of single unifacial-unipolar events (Fig. 5, n 5). Natural striking platforms (*N* = 26) are preferred over flat ones (*N* = 5), showing limited surface preparation on the cores.

**Fig. 6:**
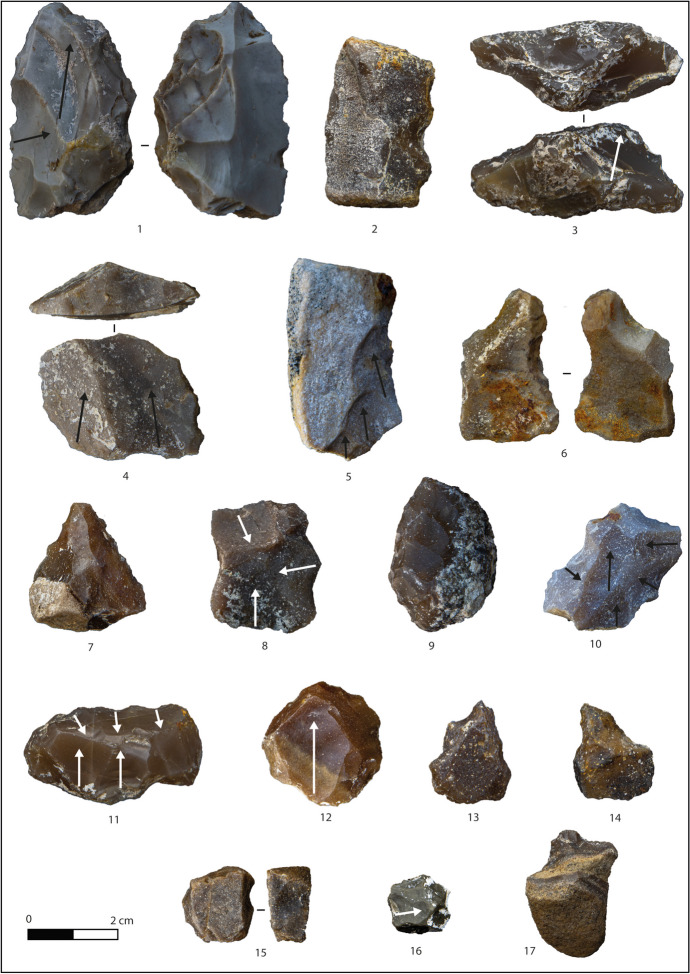
Layer G: debitage products and nodules. 1 Denticulate and point on flake of nodular chert. 2 Denticulate on nodule of flysch. 3 Double scraper on flysch flake. 4 Scraper with peripherical retouch on flysch flake. 5 Scraper on débordant flysch flake. 6 Notch with inverse retouch on nodule of flysch. 7 Point on flake with covering retouch on nodular chert. 8 Centripetal flake on flysch. 9 Scraper on débordant flysch flake. 10 Centripetal flake on flysch. 11 Retouched nodule of nodular chert: tool or core? 12 Flake on nodular chert. 13 Denticulate and point on nodule of flysch. 14 Flake on flysch. 15 Notch on nodule of flysch. 16 Flake on flysch. 17 Beak on nodule of flysch

Flakes (*N* = 58) and retouched flakes (*N* = 52) are quadrangular shaped, more developed in length than in width (Table 5; Fig. 6), with a low presence of cortical patches (19%). The presence of a back, often natural, is predominant (64%), especially on bigger flakes and tools (Fig. 6, n 5, 9). As for layer F, tools are larger than unretouched implements (Table 5). The platform analysis reveals a prevalence of flat (32%) and natural (21%) butts, then dihedral (12%), cortical (7%), facetted (5%), punctiform (4%), and linear (2%). Products without removals constitute the largest group of this layer (32%), followed by unipolar (22%), orthogonal (17%), centripetal (6%), crossed (6%), convergent (5%), and bipolar (4%). The angle of flaking has a mean value of 100°, with dihedrals butts showing a wider angle (106°). No specific relation has been detected between the removal organisation and the platform type.

A wide variety of tools can be recorded (Table 8). Scrapers are the most common, then denticulates, notches, pointed and various composite tools (Fig. 6, n 1, 3, 4, 7). The retouch is often present on more than one margin of the flakes, frequently altering their original shapes but being abrupt most of the time (Table 7). It is usually applied on the dorsal face, sometimes on the ventral or both sides (Table 7). Retouched nodules (*N* = 70) are dimensionally similar to retouched flakes (Table 5). Scrapers are the most relevant category as well, followed by denticulates and notches, which show a significant increase compared to flakes and some pointed implements (Table 8; Fig. 6, n 2, 6, 13, 15, 17). The retouch is mostly abrupt and unifacially applied on one margin (Table 7).

### Layer H

Layer H is the poorest level of Notarchirico, counting 28 lithic artefacts (Table 3) with flakes, retouched flakes, nodules, and no cores. These all exhibit smaller dimensional values than the other layers (Table 5), with retouched nodules slightly bigger than the rest. Flysch chert is the most represented raw material, followed by a small percentage of nodular chert. Flakes (*N* = 15) and tools (*N* = 4) are without cortex and show a variety of removals organisation even though opening flakes seem to prevail. The incidence of backed margin is very low (3 out of 15). Platforms are primarily flat (*N* = 7), then natural (*N* = 4), punctiform (*N* = 2), facetted (*N* = 1), and linear (*N* = 1) with a mean angle of flaking of 105°. One beak and three scrapers were recorded among the retouched flakes (Table 8), showing direct edge modifications with a marginal extension (Table 7). On the other hand, retouched nodules exclusively present an abrupt retouch to create four notches, three scrapers, and one denticulate (Table 8).

### Layer I1

Layer I1 contains 172 lithic artefacts (Table 3) mainly realised on flysch chert, even though nodular chert and radiolarite are also present in minor percentages (Table 4). Cores (*N* = 18), ranging between 20 and 80 mm (Table 5), are realised using small nodules except for one case obtained from a small fluvial pebble (30 × 35 × 0.30 mm). Cortex is present on 12 supports, equally on one or two portions.

**Fig. 7:**
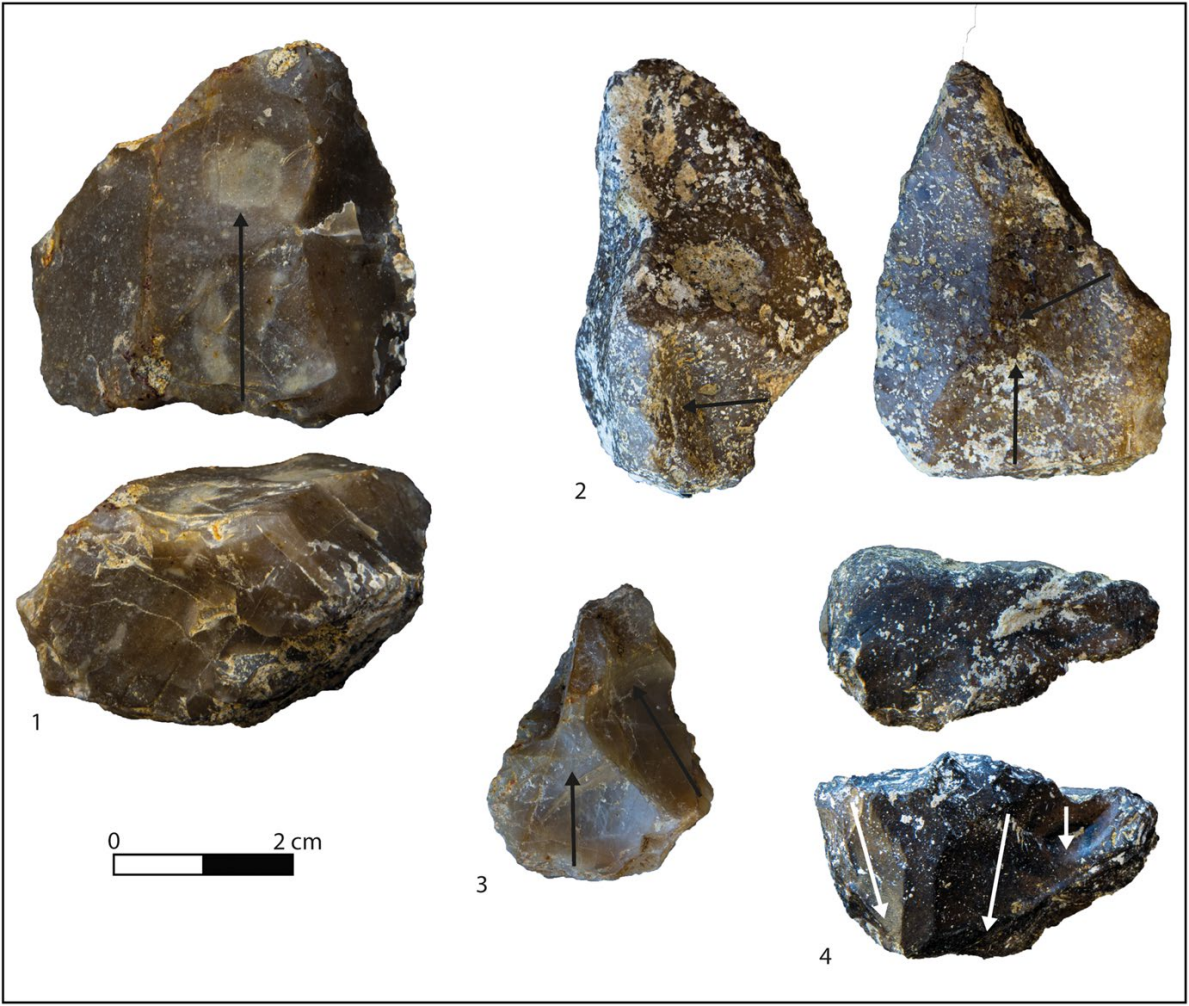
Layer I1: cores. 1 Unifacial core on nodule of flysch. 2 Bifacial core on nodule of flysch. 3 Bifacial core on nodule of flysch. 4 Semitournant core on nodule of flysch

Unifacial cores prevail (*N* = 12) and are exploited through unipolar or centripetal removals (Table 6). Centripetal ones take advantage of the natural convexities existing on some rounded nodules; they all record natural peripherical striking platforms with a flaking angle of 70° and present a maximum of three removals. Unipolar cores exhibit shorter reduction sequences, being selected for one or two removals only and exploiting almost exclusively natural platforms without cortex (Fig. 7, n 1, 4). Several cores present natural fractures and hinged removals, hinting at repetitive impacts. Bifacial cores (*N* = 8) show a mixture of removal organisation (unipolar, convergent, orthogonal, and crossed), witnessing an SSDA conception of the surfaces with a frequent inversion between the knapping surfaces and the striking platforms (Fig. 7, n 2, 3). As a result, the latter are equally natural or flat, being the outcome of the core rotation, and the angle of flaking is approximately 90°. Usually, three of four flakes were extracted from these types of supports.

Flakes (*N* = 77) and tools (*N* = 34) are always of small dimensions, ranging between 20 and 26 mm in length and 18.5 and 23 mm in width (Table 5) and quadrangular shaped. The percentage of *debitage* products bearing cortex is higher than the other layers, being present on 30% of the sample, and the cortical flakes (9%) also show an increase. The incidence of naturally backed margins is close to 50% confirming to be a recurrent technical expedient. As seen in layer G, *debitage* products without removals constitute the largest group (30%) together with unipolar (30%) and followed by orthogonal (10%), crossed (9%), and centripetal (6%). The platform analysis reveals the prevalence of flat butts (35%), natural (16%), and cortical (15%).

**Fig. 8:**
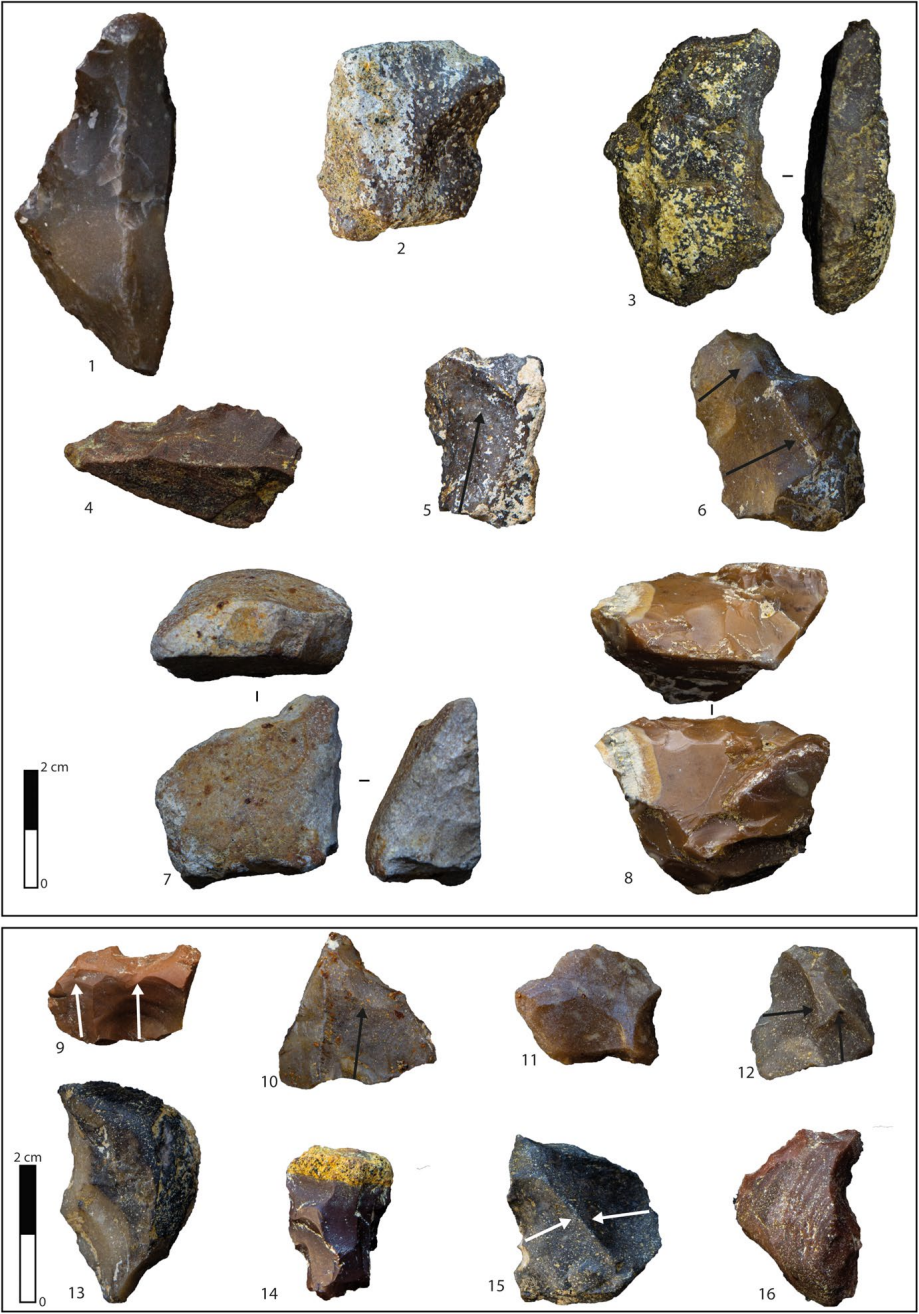
Layer I1: debitage products and nodules. 1 Composite tool with convergent retouch on flysch flake. 2 -; 3 Denticulate on flysch flake. 4 Scraper on nodule of radiolarite. 5 Débordant flake on flysch. 6 Denticulate on flake with orthogonal removals on flysch chert. 7 Convergent scraper on flysch flake. 8 Scraper on nodule of nodular chert. 9 Notch on flake of radiolarite. 10 Pointed tool on flysch flake. 11 Double ventral flake on nodular chert. 12 Flake on nodular chert. 13 Denticulate on débordant flysch flake. 14 Flake on radiolarite. 15 Flake on flysch. 16 Scraper on nodule of radiolarite

Larger flakes were selected for edge modification (Table 3). They consist of various types of scrapers, denticulates, notches, beaks, and pointed implements (Table 8; Fig. 8, n 1, 3, 6, 7, 9, 10, 13). The retouch is primarily direct and either abrupt or marginal applied on one single margin of the flakes (Table 7). Retouched nodules are slightly smaller than tools (Table 5) and are characterised by an abrupt retouch unifacially applied to produce convergent scrapers, denticulates, and notches (Tables 7 and 6; Fig. 8, n 4, 8, 16).

### Layer I2

**Fig. 9:**
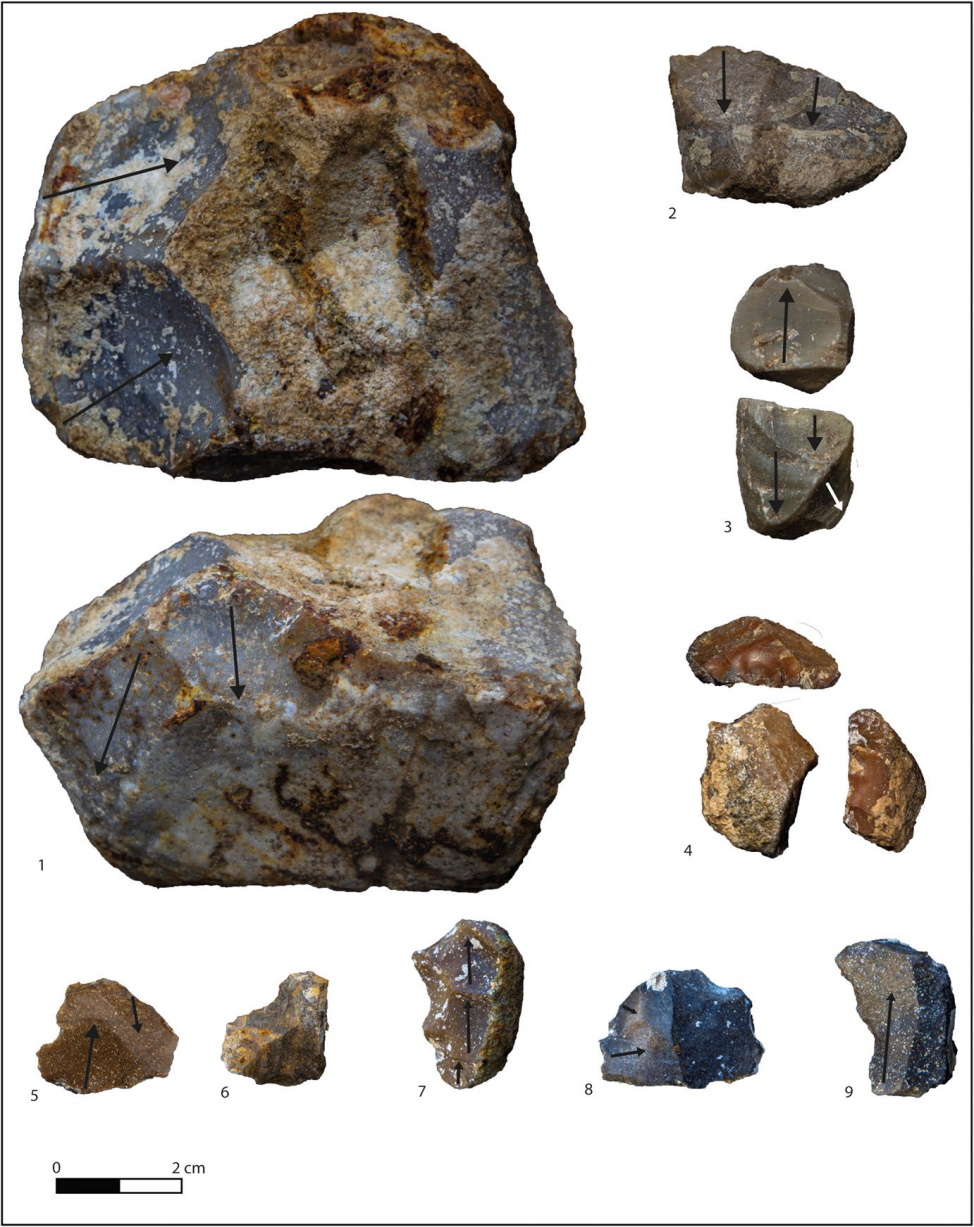
Layer I2: cores, flakes, retouched flakes, and nodules. 1 Bifacial core on large nodule of flysch. 2 Unifacial core on flysch. 3 Multifacial core on small nodule of nodular chert. 4 Scraper on nodule of nodular chert. 5 Flake on flysch. 6 Notch on flysch flake. 7 Débordant flake on flysch. 8 Flake on flysch. 9 Denticulate on débordant flysch flake

Layer I2 is the oldest level of Notarchirico and represents the beginning of the site occupation. Thirty-nine lithic artefacts were collected, including cores, flakes, tools, and retouched nodules (Table 3), mostly realised on flysch chert (Table 4). The nine cores are exploited through unipolar removals, with a mean of three per support, generally on one knapping surface in a semitournant way (Table 6; Fig. 9, n 1–3). The centripetal core takes advantage of the existing convexity of the block. Bifacial and multifacial cores witness longer reduction sequences by progressively rotating the nodule surfaces once the natural convexities are depleted. The striking platforms are natural in most cases, with the flat ones attested on bifacial and multifacial exploitations being former knapping surfaces.

Flakes and tools fit within the dimensional values of the other layers (Table 5) and are characterised by a high incidence of backed margins (65%). Despite a small sample of flakes, the removal organisation displays a mixture of unipolar (29%), centripetal (16%), crossed (13%), and orthogonal (8%) scars, while flakes without removals (20%) show a significant percentage also for this layer. Platforms are flat in half of the sample with limited presence of natural, cortical, dihedral, and punctiform butts. The absence of cortex is prevalent (63%), even if a moderate increase between the *debitage* products can be recorded. The four tools comprise two denticulates, a notch and a scraper (Table 8; Fig. 8, n 6, 9), all retouched on the dorsal face (Table 7). The retouched nodules (*N* = 6) display similar characteristics and are obtained through an abrupt modification of the margins applied on one face (Table 7; Fig. 8, n 4).

## Discussion

The new investigations conducted at the site of Notarchirico record almost 30 ka of human occupation during the initial phases of the Middle Pleistocene (695–670 ka). The technological analysis of the *debitage* products from layers F, G, H, I1, and I2 offers critical insights into hominin technological behaviour, highlighting possible similarities and discrepancies within the chrono-cultural framework of the European continent (Moncel et al., 2020b; Rineau et al., 2022).

At Notarchirico, hominins knapped different lithologies of small-sized fragmented chert nodules (30–100 mm) locally available in secondary deposits. These nodules exhibit a cubic or rounded shape with limited presence of cortex, usually located on one or two opposite edges or naturally rolled surfaces. Flysch chert is the most exploited lithotype in the stratigraphic sequence, showing a variable knapping quality according to its texture, silicification, and fracturation. The dimensional analysis of the technological categories subdivided according to the raw materials shows that flysch chert was also available in slightly larger supports than radiolarite and nodular chert (Table 9). The percentages of radiolarite and nodular chert, exhibiting a finer texture, are scarce: this is seemingly due to the actual availability in situ of these two lithotypes, but it might also reflect a systematic choice of the hominins because of dimensional values (Table 9).

**Table 9:** Size of raw material (l. = length; w. = width; t. = thickness) according to technological categories

Raw material	Flysch chert			Nodular chert			Radiolarite		
	l	w	t	l	w	t	l	w	t
Flakes	*n* = 241			*n* = 28			*n* = 4		
Min	7.7	8	2.3	5.3	7.4	1.9	14.7	12.8	6.1
Max	68.1	56.5	25.5	46.4	32.9	15.4	25.2	22.9	10.7
Mean	25.0	21.5	9.8	18.8	18.0	7.5	18.4	19.0	7.9
Ret. flakes	*n* = 73			*n* = 7			*n* = 3		
Min	9.3	10.9	4.2	15.8	12.8	5.4	14.7	12.8	6.1
Max	68.1	48	25.5	46.4	32.9	15.4	25.2	22.9	11
Mean	28.9	23.4	11.5	26.8	21.1	11.2	18.7	19.3	8.3
Ret. nodules	*n* = 100			*n* = 15			*n* = 9		
Min	12.1	12	3.9	9.8	11.1	4	11.5	14.3	5.4
Max	73.2	54	30	39.1	36.3	21.2	41.8	23.6	13
Mean	26.7	21.3	12.6	22.3	18.8	10.7	20.6	18.4	9.7

**Fig. 10:**
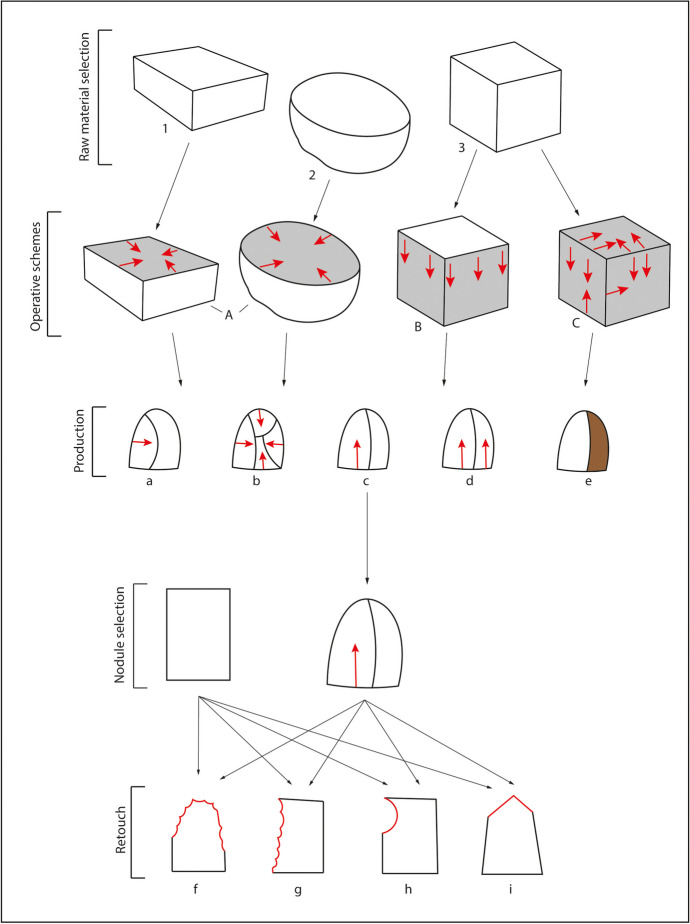
Production schemes of Notarchirico. Raw material selection: selection of nodules with rectangular (1), round (2), or cubic (3) morphologies. Operative schemes: A exploitation of one large knapping surface through a peripherical striking platform producing either orthogonal or centripetal negatives; B unipolar exploitation, eventually leading to semitournant behaviour using the natural convexities (edges and arises) of the nodules; C SSDA (systeme par surface de débitage alterné) exploitation of the cores, frequent rotation and inversion of the striking platforms and knapping surfaces. Production: typical obtained products: orthogonal flake (a), centripetal flake (b), unipolar débordant flake (c), unipolar flake (d), débordant flake without removals (e). Retouch of flakes and nodules: researched morphologies: peripheral convex retouch (f), lateral rectilinear retouch (g), notch (h), convergent/pointed retouch (i)

The morphology and size of the supports strongly influenced the technical features of the lithic assemblage of Notarchirico, which, as a result, is characterised by a homogeneous *debitage* production of small flakes and tools. The result of core technology highlights several recurrent behaviours in all layers (Fig. 10). Above all, the shape of the available nodules explains most of the hominin technical choices. However, we should also consider that technical traditions might have been developed during this process.

Cores are usually unifacially knapped through unipolar removals, sometimes producing semitournant exploitation (Fig. 10) or selected to extract one or two flakes in what might be defined as expedient behaviour. The presence of natural convexities and arrises was crucial for the nodules selection, as they were often abandoned once the morphologies were not suitable anymore, hence the high distribution of flakes without removals along the stratigraphic sequence. The *debitage* was generally conducted on the peripherical margins of the blocks, hardly altering their original volume (Fig. 10). Thus, the systematic production of *débordant* flakes could have been an efficient technical expedient to overcome the raw material constraints and speed up production. When nodules were roundly shaped, and a larger surface was available, centripetal *debitage* was also applied, exhibiting similar characteristics to unipolar cores (Fig. 10). Platforms were mostly natural without evidence of any preparation though single removals might have occurred to facilitate the obtainment of flat surfaces. This confirms a global attitude to subordinate the *debitage* to the morphological aspects of the supports. Unipolar removals characterise bifacial and multifacial cores with a frequent inversion of striking platforms and knapping surfaces pointing to an SSDA conception (Ashton et al., 1992; Forestier, 1993). The reduction sequences were more prolonged in this situation, involving a significant percentage of the core volumes but always with a maximum of two or three removals per face and limited to the exploitation of natural convexities. As previously mentioned, the incidence of flat platforms is due to the core rotation rather than the platform preparation.

Evidence of cores showing a discoid conception or structured *debitage* is scarce: only one bifacial core from layer G exhibits alternate flaking through two peripherical striking platforms. However, the *debitage* is still strongly influenced by the nodule's morphology, and there seems to be no explicit attempt to shape the surfaces regardless of their original morphologies (Santagata et al., 2020). For the time being, there is no evidence that cores were retouched or used after being discarded, though, given the sample size so far analysed and the extension of the excavation, it cannot be entirely excluded. As witnessed by several other contexts, even in the Italian Peninsula (Isernia La Pineta and Fontana Ranuccio, Ficoncella), it can be an efficient strategy—usually referred to as circularity of the reduction sequences—especially when dealing with small-sized raw materials (Aureli et al., 2016; Grimaldi et al., 2020).

The analysis of knapping strategies reveals a global mixture of unipolar, orthogonal, centripetal, bipolar, and crossed *debitage* with more or less the same distribution across the layers and a prevalence of the former. The ratio of scars per flake matches the shortness of the reduction sequences, with a mean value of 1. Only orthogonal and centripetal products present a higher proportion, given the exploitation of larger knapping surfaces. Evidence for a greater degree of complexity represented by these latter reduction sequences is, for the time, not supported by the data, indicating homogeneous yet equally complex technical behaviours modulated according to the morphological criteria. The platform distribution is dominated mainly by flat and natural butts. Still, the sporadic presence of dihedral and facetted ones indicates that preparation of the surfaces might have occurred when needed. No particular correlations were found between the platform type and the removal organisation.

If we exclude layer H, which seemingly represents a short-time occupation of the site during a mild climatic crisis (Moncel et al., 2020b; Rineau et al., 2022), the same dimensional values characterise the *debitage* products in all the layers. Flakes and tools without a cortex are predominant. An increase in cortical and partially corticated chips can be recorded in the lowest levels (I1 and I2), but it does not seem to correspond to a different economy of the raw material or technological differences. This data and the frequency of products showing no removals confirm the scarcity of cortical remains on nodules, including their intentional selection and the massive exploitation of natural surfaces. Backed flakes are also abundant, attested on at least 50% of the lithic assemblage for each layer and often opposed to a cutting margin. Retouched tools are always realised on bigger implements and are characterised by scrapers, denticulates, notches, beaks, and pointed flakes, revealing various types and, seemingly, functions. Their distribution is uniform across the stratigraphy, with layers G and I1, the richest of the site, exhibiting a greater diversity. The retouch can vary, applied on a single margin or peripherally and frequently altering the original shape of the support to obtain specific morphologies. The angle of retouch is mainly included between 60° when it is marginal and located on thin edges and 80° when it is abrupt. Regardless of the selected layer, this pattern is constant along the stratigraphic sequence. No relations were detected between the flakes chosen for the retouch and the knapping strategies.

Hominins also selected many small nodules of the same size as tools to be retouched. These nodules show a cubic/rectangular shape, hardly exhibiting natural cutting edges and with scarce attestation of the cortex. Naturally backed margins on the nodules could have played an essential role in their selection, representing a possible prehensile part, opposed to the modified edge. Consequently, the retouch was almost exclusively abrupt on one face to obtain scrapers, denticulates, beaks, and notches. It may be safe to argue that the role of tools and retouched nodules was the same since the latter represents a valid substitute for retouched flakes, even from a typological point of view. The dimensional values confirm this aspect showing an intentional selection of supports with a specific length. The analysis of the raw material economy provided the same result as the *debitage* production. It is essential to point out that retouched nodules and flakes do not show a second phase of reshaping or recycling, which could indicate a single-time use and short lifespan of these products. Several retouch flakes have been found in different layers, which, together with all the gathered data, indicate that the lithic objects were knapped, used, and abandoned in the same area.

The possibility of nodules being used as passive supports/cores to extract small-sized flakes is still open for the time being. On larger nodules, there is evidence of removals dimensionally comparable to the end products with a suitable flaking angle (close to 90°). Further investigations are required to clarify the possible existence of this behaviour; however, the ambivalence of the concepts of *debitage* and *façonnage*, which seems to affect these chronological phases—particularly in contexts characterised by raw materials of small dimensions like Isernia La Pineta, Ficoncella, Fontana Ranuccio, and Soucy—might be a crucial technological trait to track down and a possible marker of innovation (Aureli et al., 2016; Grimaldi et al., 2020; Lhomme, 2007).

Ultimately, the analysis of the core does not reveal remarkably structured reduction sequences from a morphological and conceptual point of view. Nevertheless, this does not mean that the lithic assemblage of Notarchirico lacks complexity from a methodological perspective. The systematic use of retouch to shape the original morphologies of the small available supports according to the production goals and the exploitation of nodules demonstrate a skilful adaptation to the raw material by these hominins, balancing out the qualitative and dimensional constraints and allowing them to obtain a great variety of products. Besides, core management homogeneity along the stratigraphic sequence highlights a behavioural response of these hominins to approach this type of raw material that gradually becomes systematic and is assimilated within the methodological process.

Following this idea, it is remarkable noticing that the Italian Peninsula is noted for numerous sites (Notarchirico, Isernia La Pineta, Loreto, Ficoncella, Cimitero di Atella, Fontana Ranuccio, among others; Abruzzese et al., 2016; Aureli et al., 2016; Gallotti & Peretto, 2015; Grimaldi et al., 2020; Lefèvre et al., 2010; Muttillo et al., 2021) spanning from the beginning of the Middle Pleistocene (MIS 19) to approximately 400 ka (MIS 11) exhibiting a massive production of small-sized flakes and retouched tools—sometimes associated with the production of handaxes as in the case of Cimitero di Atella and Notarchirico itself. This aspect has often led the scientific community to identify a potential pattern in the Italian Peninsula originating from the raw material’s availability and seemingly becoming cultural and behavioural (Gallotti & Peretto, 2015; Muttillo et al., 2021).

Various pebbles and large cutting tools also characterise the site of Notarchirico (Moncel et al., 2020b), showing a sharp increase in the uppermost portion of the sequence simultaneously with the appearance of the earliest bifaces of the site so far (layers G and F; Table 2). Pebble and large cutting tools are described as poorly standardised from a morphological point of view (Moncel et al., 2020b); they exhibit a broad diversification with unifacial, bifacial, and trifacial retouch, partially altering the original shape of the supports when possible and consistently taking advantage of the available natural convexities recalling the pattern seen for the *debitage* products and retouched nodules. On the other hand, the six bifaces are reported to show skilful management of the bifacial and bilateral symmetry—with peripherical removals and a final retouch phase to regularise the cutting edges—fitting into the Acheulean paradigm that begins to emerge at the onset of the Middle Pleistocene within the European continent and of which Notarchirico represent one of the earliest evidence (Moncel et al., 2019).

The presence of bifaces is commonly associated with technological—and cognitive—shifts in the lithic assemblages where they have been found, which, in this case, the *debitage* production does not seem to reflect. The analysis of all the layers witnesses an—alleged—abrupt appearance of these items starting from layer G but does not reveal a change in the degree of complexity of core technologies and flake production—together with nodule fabrication—whose characteristics and conception are somewhat similar to the heavy-duty components remaining homogeneous along the considered layers (Rineau et al., 2022). It is plausible that the absence of bifaces in layers H, I1, and I2 could be due to the excavation's size—as already proposed in other works (Moncel et al., 2020b; Rineau et al., 2022)—despite being investigated on the same area of layers F and G but might also reflect a change in the site’s role and function over time. On the other hand, layers F and G are indeed the richest and most diversified quantitatively and typologically speaking, which could suggest an actual shift in the modalities of the occupation or the conducted activities.

Could we truly assign to the bifaces this role of cultural marker/complexity changer in the site of Notarchirico, which might already be present in the lowest levels? If we assume that (1) the hominins’ adaptive response to the exploitation of identical raw materials of small morphologies becomes systematical from the bottom of the stratigraphic sequence to which handaxes are later integrated, (2) aspects such as the spatial mobility within the site and its function may vary over time, and (3) the absence/presence of the bifaces is seemingly not due to an actual shift from a complexity-free context to a more complex one—as the technological analysis seems to suggest—then other behavioural variables should also be considered as additional proxies of possible “cultural” and evolutionary changes (Binford & Binford, 1966; Henrich, 2015; Davis & Ashton, 2019; Pargeter et al., 2019). For instance, the possible exploitation of organic material (i.e. bones) to compensate the lack of raw materials of large dimensions.

The archeozoological and functional data available do not record discrepancies along the stratigraphic sequence in the exploitation of faunal remains and worked materials, though the data are partial and still being processed (Moncel et al., 2020b). The use-wear analysis proved that the site was not exclusively cutting-oriented, especially from the basal portion of the sequence—which might have explained a delayed introduction of bifaces—with evidence of wood and plant processing preserved on the margins of the *debitage* products. This aspect might contribute to the potential continuity of the site concerning the practised activities and following the substantial homogeneity depicted by the lithic assemblage, portraying Notarchirico as a multi-functional context with recurrent continuous occupations during both glacial and interglacial phases (Moncel et al., 2020b; Rineau et al., 2022).

Moving onto the European chrono-cultural framework, Notarchirico provides one of the earliest examples of the Acheulean techno-complex together with the French sites of La Noira (700 ka) and Moulin Quignon (Moncel et al., 2020a; 2021c). In these contexts, some similarities exist within the degree of complexity of bifacial assemblages, exhibiting the complete ability to manage bifacial and bilateral symmetry, other than the presence of heavy-duty implements and large cutting tools; however, both French contexts show the use of soft hammer percussion for the final shaping of the bifaces which Notarchirico does not. The use-wear analysis of La Noira also indicates the presence of diversified activities and exploited materials such as cutting meat, wood and plant processing, bone-working, and engraving, similar to Notarchirico (Hardy et al., 2018). Unlike the latter, however, La Noira shows traits of knapping innovations, primarily when centripetal cores are addressed, highlighting more structured and organised reduction sequences capable of subordinating the raw material morphologies, hierarchising the surfaces, and closer to the bifacial conception of *shaping* (Moncel et al., 2021a). It is essential to underline that the raw material employed at La Noira is composed of large slabs of fine texture that could have granted dimensional and technical advantages to the hominins.

Aside from this, the core and flake production of Notarchirico fits within the “small-sized” flakes contexts of the Middle Pleistocene, such as Isernia La Pineta (590 ka), Ficoncella (500 ka), and Atapuerca TD6 (800 ka), whose lithic assemblages resemble the Mode 1 *debitage* (Aureli et al., 2016; Gallotti & Peretto, 2015; Mosquera et al., 2018). In these contexts, characterised by the massive production of small flakes and tools on local raw materials, the absence of bifacial implements is reported. It is unclear whether this is due to block's dimension, cultural substratum—which has often led the scientific community to exclude them from the Acheulean techno-complex—functional reasons or size of the excavations area. Recent works from Atapuerca TD6 pointed out that the absence of handaxes at the site is due to an actual absence of the bifacial concept implying a systematic technological choice of the hominins rather than issued from the raw materials availability (Lombao et al., 2022; Mosquera et al., 2018).

Nonetheless, Notarchirico stands in a crucial spot because of its geographic location, close to the other Italian Lower Palaeolithic sites, and as an alternative entry route to Europe for the African migratory fluxes together with Gibraltar and Levantine corridor (Abbate & Sagri, 2012). Besides, the climatic background of the Italian peninsula at the beginning of the Middle Pleistocene makes it a sort of shelter area, occupied during both interglacial and glacial stages—due to the moderate climatic variations—as confirmed by the radiometric datings, and thus offered continuous frequentation (Bertini, 2003; Pereira et al., 2018). In the end, the mixed features of Notarchirico’s lithic assemblage, halfway in between “persistency” (cores and flake production) and innovation (bifacial tools), make it a cornerstone in understanding the different behavioural responses of the hominins. The data so far gathered raised questions about whether the emergence of handaxes is due to an in situ evolution or an allochthonous introduction, with a constant reminder that the function and occupation of a site strongly influence the material culture and the human response.

## Conclusion

The new investigations conducted at the site of Notarchirico pushed back the emergence of the bifaces within the Italian Peninsula to 680 ka (layer G), aligning with the recent discoveries of the French sites of La Noira and Moulin Quignon and attesting to a homogeneous arrival of the Acheulean techno-complex in Europe during the interglacial 17. The site features a prolonged human occupation during stages 17 and 16 of the Middle Pleistocene, being a unicum in the European Lower Palaeolithic and acting as an ecological niche for faunal and human groups. Hominins of Notarchirico took advantage for a long time of the paleo-channels to exploit the presence of water, animal carcasses, woods, plants, and lithic raw materials (limestone and various type of chert). The archaeological data suggest recurrent and stable occupation of the hominins across all the layers of the site, whose activities are diversified, including cutting meat, wood and plant processing, and bone-working, hinting at a “domestic” configuration of Notarchirico—as also proposed for La Noira—with seemingly high mobility over large areas (sitewide). The analysis of the lithic assemblage shows the exploitation of locally collected chert and limestone to realise various large-sized tools (bifaces, cutting tools, pebble tools, etc.) and small flakes and tools (scrapers, denticulates, notches, and pointed implements). Hominins also selected small-sized chert nodules directly to be retouched, functioning as an alternative to retouched flakes. The technological behaviour proved to be homogeneous from the bottom to the top of the newly investigated sequence (layers I2, I1, H, G, and F), focusing on *debitage* production and pebble tools. At the same time, the presence of bifaces is attested only from layers G and F.

This raises questions about whether the introduction of this particular technology is due to an abrupt arrival of new populations—and behaviours—or to a local evolution as an adaptive response to environmental pressures. It should also be considered that a change in the site function might have occurred, leading to the integration of bifaces within the toolkit of the hominins of Notarchirico, adding to an already diversified lithic corpus comprising *debitage* production and heavy-duty components. To conclude, Notarchirico is characterised by a substantial homogeneity of techno-economic behaviours, covering both an interglacial and glacial phase in southern Europe where the climatic variations were low, which, allegedly, only the presence of bifaces seems to break, acting as an element of innovation and connoting the site as in between cultural innovation and continuity.

## Data Availability

Not applicable.
